# Cardiac Tamponade Due to Diffuse Large B-cell Lymphoma/Plasmablastic Lymphoma in an Immunocompetent Patient

**DOI:** 10.7759/cureus.72891

**Published:** 2024-11-02

**Authors:** Kartikeya Kaul, Maria A Kelley, Umang Patel, Patrick May, Thanh Nguyen

**Affiliations:** 1 Internal Medicine, St Mary Medical Center, Langhorne, USA

**Keywords:** cardiac tamponade, cardio-oncology, extraoral plasmablastic lymphoma, lymphoma, non-hodgkins lymphoma

## Abstract

Plasmablastic lymphoma (PBL) is a rare and aggressive subtype of diffuse large B-cell lymphoma, present predominantly in immunosuppressed individuals, particularly with human immunodeficiency virus (HIV) or Epstein-Barr virus (EBV) infection. Although the incidence of HIV-positive vs HIV-negative PBL is unknown, a literature review vastly associates it with immunocompromised status. Primarily seen in the male population in approximately 75% of reported cases, PBL has a well-established clinical presentation of one or two extra-nodal masses, generally in the oral cavity and gastrointestinal tract. We present a case of a 93-year-old female without any evidence of HIV infection or extra-nodal mass who presented with shortness of breath due to a large malignant pericardial effusion that evolved into a tamponade. Plasmablastic lymphoma is one of the few reported in HIV-negative individuals, and its unique presentation as a pericardial effusion without extranodal mass makes its suspicion less apparent. However, awareness of this entity, comprehensive immunohistochemistry, and most importantly, correlation with clinical presentation is the cornerstone to establishing a correct diagnosis.

## Introduction

Plasmablastic lymphoma (PBL) is a rare and aggressive subtype of diffuse large B-cell lymphoma, present predominantly in immunosuppressed individuals, particularly with human immunodeficiency virus (HIV), Epstein-Barr virus (EBV), and HHV8 infection. Although the incidence of HIV-positive vs HIV-negative Plasmablastic lymphoma is unknown, a literature review vastly associates it with immunocompromised status. Primarily seen in the male population in approximately 75% of reported cases, plasmablastic lymphoma has a well-established clinical presentation of one or two extra-nodal masses, generally in the oral cavity and gastrointestinal tract.

## Case presentation

A 93-year-old female with a medical history of hypertension and hyperlipidemia presented to the Emergency Department for lightheadedness, dizziness, changes in vision, bilateral lower extremity paresthesia, and diarrhea for about 24 hours. In the Emergency Department, a CT brain was unremarkable and she was treated with IV fluids, admitted for observation, and discharged. Four days later, symptoms progressed in severity prompting her to report to the Emergency Department on a separate occasion with shortness of breath, 14 lbs weight gain, decreased appetite, and persistent diarrhea. Lab results are listed in Table 1.

**Table 1:** Laboratory results of the patient MCV: mean corpuscular volume; AST: aspartate aminotransferase; SGOT: serum glutamic-oxaloacetic transaminase; ALT: alanine aminotransferase; SGPT: serum glutamic-pyruvic transaminase

Lab Component	Reported Value	Normal Range
WBC	6.6	4.3-10.5 K/mcL
Hemoglobin	10.1	11.6-15.0 g/dL
MCV	99.1	80-96 FL
Platelets	224	150-400 K/mcL
Sodium	129	136-145 mmol/L
Potassium	3.6	3.5-5.1 mmol/L
Creatinine	1.49	0.55-1.02 mg/dL
AST (SGOT)	95	15-37 unit/L
ALT (SGPT)	131	7-52 unit/L
Alkaline Phosphatase	103	34-104 unit/L
Total Protein	6.3	6.4-8.2 g/dL
Albumin	4	3.5-5.2 g/dL
Globulin, Total	2.3	2.6-3.5 g/dL

The physical examination was unremarkable, with no lymphadenopathy or splenomegaly, and the patient was hemodynamically stable and saturating 97% of room air.

A CT scan of the chest showed moderate to large pericardial effusion with small bilateral pleural effusions, atelectatic changes at the lung bases with small infiltrates, as well as a small volume of upper abdominal ascites. A subsequent echocardiogram described a large pericardial effusion. The patient became hypotensive and bradycardic concerning cardiac tamponade and a rapid pericardiocentesis was performed, removing 500 ml of dark bloody fluid and resolving the acute presentation. Pericardial fluid showed 1,392,426 RBC/mm^3^, 29,872 nucleated cells/mm^3 ^consisting of 5% neutrophils, 7% lymphocytes, 7% monocytes/macrophages, and 81% other cells. Additional laboratory testing was ordered to determine the etiology of the effusion, antinuclear antibody (ANA) was elevated showing an antibody titer of 1:80, and rheumatoid factor (RF), antineutrophil cytoplasmic antibodies (ANCA), myeloperoxidase, and proteinase-3 were all negative. Pericardial fluid cytology showed high-grade hematopoietic neoplasm with large-cell plasmablastic features. 

Microscopy demonstrated multinucleated plasma cells and numerous mitotic features, CD5, CD45, and CD138 were positive, the Ki-67 proliferation marker was >95%, and flow cytometry showed 77.9% of plasma cell/plasmacytoid B-cell, which showed cytoplasmic light chain restriction with CD38 + CD45+, CD138+, as well as dim expression of DC117. CD19, CD20, and PAX-5 were negative. Urine and serum protein electrophoresis revealed a monoclonal gammopathy of the IgA Kappa type. Immunophenotyping results and the lack of favorable features of plasma cell myeloma assisted in establishing a very likely diagnosis of plasmablastic lymphoma. Subsequent chest CT angio revealed multiple paravertebral masses and enhanced axillary lymph nodes. The patient declined the biopsy as well as curative treatment based on unfortunately low survival rates and an aggressive course of disease. The patient proceeded with comfort measures and passed away a few months after the initial diagnosis.

**Figure 1:**
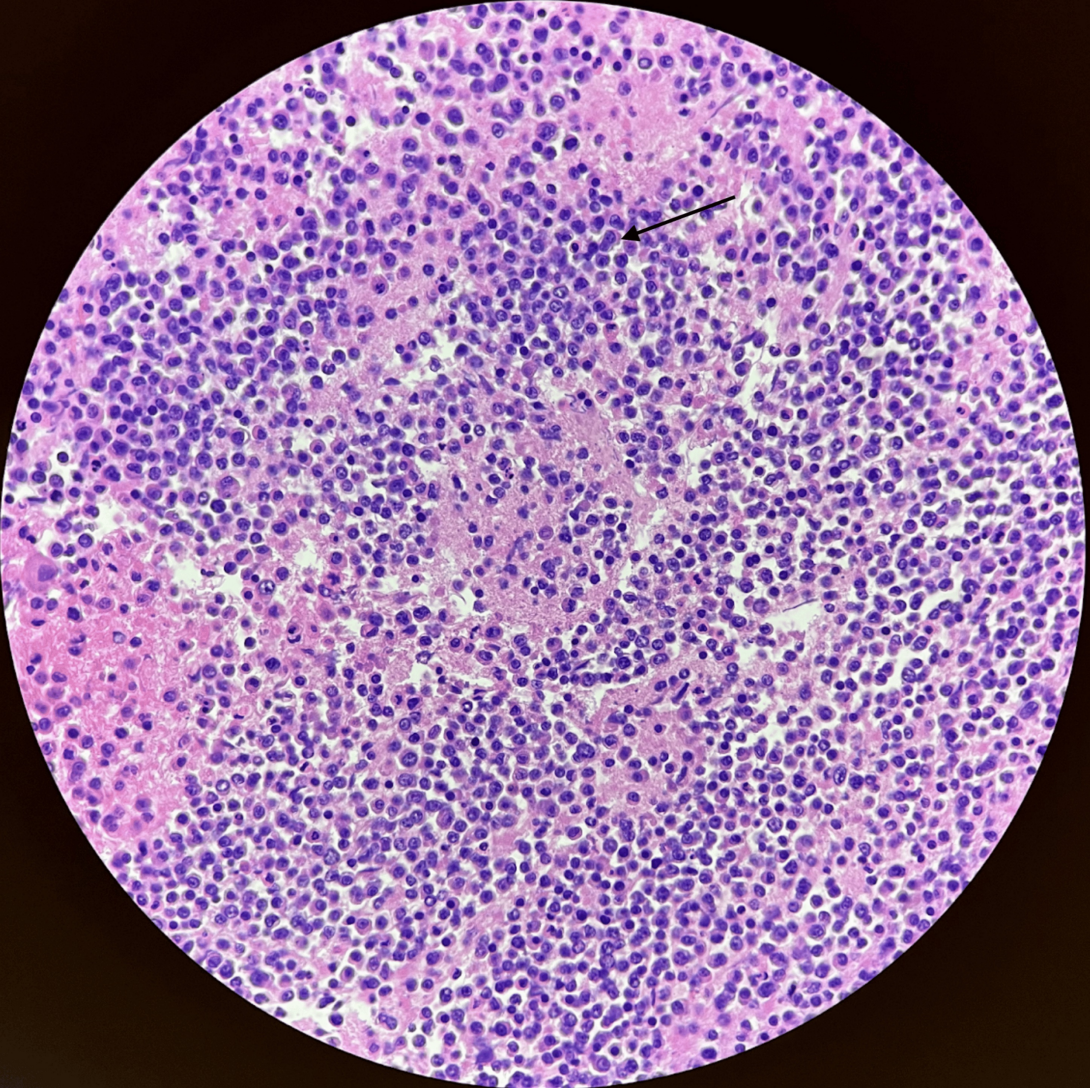
H&E (hematoxylin and eosin) stain at 40x magnification: numerous multinucleated plasma cells with several mitotic features (black arrow)

Histopathological studies were done on pericardial fluid. H&E staining (Figure 1) demonstrated multinucleated plasma cells and numerous mitotic features, as well as CD5 and CD138 positivity. Flow cytometry showed 77.9% of plasma cell/ plasmacytoid B-cell, which showed cytoplasmatic light chain restriction. SPEP/UPEP revealed a monoclonal gammopathy of the IgA Kappa type.

## Discussion

Plasmablastic lymphoma is an extremely rare and aggressive subtype of diffuse, large B-cell lymphoma. It encompasses less than 1% of all non-Hodgkin lymphomas [1], and due to its overlapping morphological and immunophenotypic features with other lymphomas with plasmablastic differentiation especially plasmablastic myeloma, its diagnosis must consider the entire clinical scenario and radiological and other laboratory findings [2]. In addition, plasmablastic lymphoma has a strong association with immunodeficiencies, especially HIV and EBV, as well as post-transplant iatrogenic immunosuppression, less commonly arising from previous autoimmune disease or lymphoproliferative disorder, with only a few cases reported in immunocompetent individuals [3].

It presents predominantly in males in about 75% of cases and, interestingly, women diagnosed with plasmablastic lymphoma usually have a previous autoimmune, lymphoproliferative disorder or show an immunocompetent status [2]. The majority of patients present with a fast-growing extranodal mass mainly in mucosal tissue [4-6]. It is located primarily in the oral cavity and, subsequently, the gastrointestinal tract, although it has been reported as nodal involvement mainly in post-transplant iatrogenic immunosuppression [7]. The diagnosis and differentiation of plasmablastic lymphoma enclose histology and cytology studies, immunophenotyping, genetic testing, radiology, including CT scans and positron emission tomography (PET) scans, and perhaps most importantly, clinical presentation. Concerning the pathological features of plasmablastic lymphoma, it presents as hypercellular specimens, high-grade neoplasia with large immunoblast or plasma cells, and occasionally, multinucleated and anaplastic forms. Plasmablastic lymphoma immunophenotypically resembles plasma cell neoplasia and usually expresses CD38, CD138, IRF/MUM1, PRDMI/BLIPM1, XBP1, and cytoplasmic immunoglobulin light chain restriction [2]. These cells are negative for B-cell markers CD19, CD20, and PAX-5; however, they can show dim positivity for CD45, and in some cases, we can find positive T-cell markers like CD2 or CD4, loss of regular plasma cell markers, such as CD27, CD81, and the Ki-65 proliferation index is, without exception, >70% [3]. Essential adjuvants to diagnosis are HIV, EBV, HHV-8 serological studies, serum and urine electrophoresis, and molecular and genetic testing often reveals MYC amplifications and has demonstrated that plasmablastic lymphoma is genetically more closely related to diffuse large B-cell lymphoma (DLBCL) than myeloma [3,8-10].

Unfortunately, the prognosis is generally poor due to the aggressive course of the disease. Research shows the median survival rate is 6-19 months, and HIV status doesn't seem to make a difference in the prognosis. Based on our literature review, this case of plasmablastic lymphoma is one of the few reported in HIV-negative individuals, and its unique presentation as a pericardial effusion without an extranodal mass makes its suspicion less apparent. However, awareness of this entity, comprehensive immunohistochemistry, and most importantly, correlation with clinical presentation is the cornerstone to establishing a correct diagnosis.

## Conclusions

Plasmablastic lymphoma (PBL) is a rare and aggressive subtype of diffuse large B-cell lymphoma, present predominantly in immunosuppressed individuals, particularly with human immunodeficiency virus (HIV) or Epstein-Barr virus (EBV) infection. Based on our literature review, this case of plasmablastic lymphoma is one of the few reported in HIV-negative individuals, and its unique presentation as a pericardial effusion without an extranodal mass makes it less suspicious. However, awareness of this entity, comprehensive immunohistochemistry, and most importantly, correlation with clinical presentation is the cornerstone to establishing the correct diagnosis.
